# Protocol design and current status of CLIVIT: a randomized controlled multicenter relevance trial comparing clips versus ligatures in thyroid surgery

**DOI:** 10.1186/1745-6215-7-27

**Published:** 2006-09-01

**Authors:** CM Seiler, BE Fröhlich, JA Veit, E Gazyakan, MN Wente, C Wollermann, A Deckert, S Witte, N Victor, MW Buchler, HP Knaebel

**Affiliations:** 1 Study Center of the German Surgical Society (SDGC), University of Heidelberg, Germany; 2 Institute of Medical Biometrics and Informatics (IMBI), University of Heidelberg, Germany; 3 Department of General-, Visceral-, Trauma Surgery, University of Heidelberg, Germany

## Abstract

**Background:**

Annually, more than 90000 surgical procedures of the thyroid gland are performed in Germany. Strategies aimed at reducing the duration of the surgical procedure are relevant to patients and the health care system especially in the context of reducing costs. However, new techniques for quick and safe hemostasis have to be tested in clinically relevance randomized controlled trials before a general recommendation can be given. The current standard for occlusion of blood vessels in thyroid surgery is ligatures. Vascular clips may be a safe alternative but have not been investigated in a large RCT.

**Methods/design:**

CLIVIT (Clips versus Ligatures in Thyroid Surgery) is an investigator initiated, multicenter, patient-blinded, two-group parallel relevance randomized controlled trial designed by the Study Center of the German Surgical Society. Patients scheduled for elective resection of at least two third of the gland for benign thyroid disease are eligible for participation. After surgical exploration patients are randomized intraoperatively into either the conventional ligature group, or into the clip group. The primary objective is to test for a relevant reduction in operating time (at least 15 min) when using the clip technique. Since April 2004, 121 of the totally required 420 patients were randomized in five centers.

**Discussion:**

As in all trials the different forms of bias have to be considered, and as in this case, a surgical trial, the role of surgical expertise plays a key role, and will be documented and analyzed separately. This is the first randomized controlled multicenter relevance trial to compare different vessel occlusion techniques in thyroid surgery with adequate power and other detailed information about the design as well as framework. If significant, the results might be generalized and may change the current surgical practice.

## Background

Annually, a total of more than 90.000 surgical thyroid procedures are performed in surgical departments throughout Germany [1]. The prevalence of the endemic goiter in the adult population ranges between 20% and 30% in this iodine deficiency area [2]. Today, thyroid surgery is a highly standardized procedure with low morbidity and mortality. Several techniques of occluding vessels, besides the classic ligatures, are currently available. The clamp and tie technique is still the most frequently used standard, although it requires several steps which in addition are time-consuming. An alternative technique is the application of vascular clips that have proven to be fast, effective and safe for achieving hemostasis in different surgical areas [3,4]. In thyroid surgery, however, no randomized controlled trial (RCT) has so far been performed comparing vascular clips with the conventional clamp and tie technique. Only ultrasonically activated shears were tested against the conventional technique in a RCT and found to be a suitable device in total thyroidectomies and lobectomies [5,6]. However, Voutilainen et al. (2000) stated that the advantage of the ultrasonically activated shears in thyroid surgery is probably of less importance for surgeons who achieve good results with vascular clips.

### Aim of the study

Besides achieving the best surgical outcome (low morbidity and mortality as well as absence of recurrence), operating time is an important factor for thyroid surgeons [7-10]). A reduction in operating time is not only beneficial for the patients, due to reduction of the risk associated with the time of general anaesthesia, but also for the health care system, because every minute of operating time saves approximately 8–11 € [6,11]. Hence, there is a large interest in performing surgery that is not only safe, but also fast and cost effective. Especially in centers with more than 60 – 100 thyroidectomies annually, the introduction of methods which potentially may reduce operating time, will significantly increase the level of effectiveness [12].

### Need for this study

To date, no clear scientific recommendation exists in regard to the available blood vessel ligation techniques in thyroid surgery. A systematic review comparing different blood vessel ligation techniques has not yet been performed. The literature on this topic shows several deficits thus reducing its internal and external validity. Main problems are underpowered sample sizes [6,13], missing data in regard to the surgical technique [5], lack of standardization of the various surgical techniques [5] and of homogeneity of the used study designs [6].

Therefore a randomized controlled multicenter, patient-blinded, two-group parallel relevance study under standardized conditions is required in order to achieve high internal as well as external validity. This would allow the results to be generalized for thyroid surgery and also may have implications for other surgical settings. This study is one of the first studies designed and organized by the Study Center of the German Surgical Society (SDGC). Besides investigating the primary hypothesis, this study was also started with the aim of developing a nationwide structure for randomized trials using a common and surgical relevant procedure [14].

This article describes the design and current status of the CLIVIT trial to publicly lay open the assumptions that have been made at the beginning of the trial. According to Chan et al. (2004) it is not uncommon that primary endpoints are changed during the conduction of a clinical trial leading to substantial protocol violations and thereby misleading the medical community [15]. A protocol publication might help to prevent this. Surgical trials are still inferior in their internal validity compared to pharmacological studies [16]. Especially the clear description of concealed allocation sequences, interventions and assessment of surgeons' experience, safety issues and outcome evaluation are of interest.

## Methods/design

### Trial population

The major eligibility criteria is a benign disease of the thyroid gland – except Graves disease – scheduled for elective (a) hemithyroidectomy and subtotal resection of the other side, (b) near total or (c) total thyroidectomy. Malignant diseases of the thyroid gland, hyperparathyroidism, re-operations and Graves disease were excluded from the study for they do have a higher risk for damage of the recurrent laryngeal nerve in surgery and/or they are not standard operations in smaller hospitals [17]. Detailed inclusion and exclusion criteria are specified in Table 1. Patients are screened consecutively for eligibility in all participating centers after approval of the study protocol by the local ethics committee. A contract has been signed by the SDGC and the participating hospitals for correct conduction of the trial according to Good Clinical Practice. All participating surgeons performing the interventions have been instructed by detailed manuals and the clips as well as their reusable applicators are provided.

**Table 1:** CLIVIT trial: study inclusion and exclusion criteria

INCLUSION CRITERIA	EXCLUSION CRITERIA
At study enrollment: • Age equal or greater than 18 years • Expected survival time more than 12 months • Patients with benign diseases of the thyroid gland scheduled for elective surgery • Euthyroid metabolism (normal level of TSH or T_3_/T_4_) • Normal function of the vocal cords • Informed consent	• Malignant disease or highly suspicion for malignancy (clinical and imaging evidence) • Nerve palsy • Graves' Disease • Coagulopathy • Current immunosuppressive therapy • Chemotherapy within 2 weeks before operation • Radiotherapy completed no longer than 8 weeks before operation • Severe psychiatric or neurologic disease • Drug- and/or alcohol-abuse • Participation in another intervention-trial with interference of intervention and outcome • Inability to follow the instructions given by the investigator or the telephone interviewer • Lack of compliance
At the end of surgical exploration: • Suitable for at least 2/3 resection of the thyroid	

### Clinical sites and safety aspects

Sites are selected according to their experience in thyroid surgery and willingness to adhere to the clinical trial protocol. The surgical expertise of each participating surgeon is documented and taken into account during the analysis of the trial data. The participating centers are listed at the end of this paper.

For safety reasons concomitant medication and medical history are checked prior to operation. Patients at higher risk for complications (e.g. bleeding disorder, history of neck operation) are excluded from the study. All serious adverse events (SAE) will be reported to the principal investigator and the leading ethics committee. For the safety analysis the incidence of adverse events (AE) and SAE will be analyzed. Patients may withdraw from the study at any time either at their own request or at the request of the principal investigator.

### Interventions

A standardized surgical approach to the thyroid gland will be performed in both groups:

The skin is cut with a conventional scalpel (Kocher's incision). Subcutaneous layer and platysma are cut with the electric cautery. After splitting cervical fascia, the infrahyoideal muscles are set aside with retractors. Cervical muscles should be cut only exceptionally. The thyroid gland is then completely exposed in order to define the extent of the required surgical procedure. If the findings are in accordance with the trial inclusion criteria, randomization is performed. Regardless of the randomization result the procedure always starts with preparation and ligation of the upper pole vessels on the side of the hemithyroidectomy (Novafil^® ^3-0 or equivalent suture material). At the moment of dividing the upper pole vessels the time is taken and the operation then continues according to the regulations of the specified randomization group.

### Clip Group

In the Clip Group each vessel will be occluded by application of one medium size clip close to the thyroid and two medium-size clips distal to the thyroid using the BBD-Aesculap Challenger^® ^clip applicator (BBD-Aesculap, Tuttlingen, Germany).

### Ligature Group

In the ligature group each vessel will be occluded by manual ligature (Vicryl^® ^3-0 or equivalent suture material) using the square-knot technique and divided with scissors after ligation.

### Both groups

The bipolar diathermy as an additional tool is allowed in both study groups. Time measurement stops as soon as the thyroid gland is removed. Following the resection, the capsule is sutured with an inverted continuous suture technique (PDS^® ^4-0 or equivalent suture material). After hemostasis uni- or bilateral drains (10 French sized Redons-drains) are placed into the resection area. Cervical muscles in the mid-line and platysma are adapted with conventional interrupted sutures. The operation is completed with intracutaneous sutures and sterile wound dressing.

### Objectives and outcomes

The primary objective of this study is to test whether there is a relevant reduction in operating time (>15 min), using the clip vessel occluding technique versus the conventional clamp-tie. Secondary objectives are divided into surgical and non-surgical categories. In the surgical category there are the following points: total duration of operation (skin incision until suture), weight of specimen, amount of postoperative bleeding, frequency of reoperation due to bleeding, recurrent laryngeal nerve paralysis (temporary or irreversible – pre- and postoperative assessment of vocal cord function by ENT-specialist), wound infection rate and rate of impaired parathyroid gland function. The non-surgical category consists of duration of postoperative hospital stay. To assess the relevance of the chosen endpoints, the following ten aspects have to be ranked from "most important" to "least important" by patients and surgeons (only once): Intraoperative complications, postoperative complications, length of hospital stay, voice function, dysphagia, death, postoperative pain, postoperative fatigue, convalescence of complete physical maximum resilience and cosmetic result [18].

### Sample size

A relevant difference between the two operations is defined as greater than 15 minutes. The expected difference is 20 minutes. Analysis of retrospective data showed a standard deviation of about 27 minutes for the whole operation. For the primary endpoint a percentage of 70% of the whole operation time is assumed. If this percentage were exactly 70% for every operation, standard deviation for the primary endpoint would be 0.7*27 min = 18.9 minutes. Since this relation will probably not be so strict, the standard deviation for the primary endpoint is estimated to be higher. Thus, a standard deviation of 20 minutes is assumed. The α level is set to 0.05; therefore, 200 patients per group are required to obtain a power of 80% (β = 0.2) using a simple one-sided t-test for two groups with a shifted null-hypothesis. Due to the fact that no interaction is expected regarding surgeon experience and treatment group, the sample size calculation is also valid for the ANOVA model of the primary analysis. In order to incorporate an expected early dropout rate of 5%, a total of 420 patients is required.

### Randomization and blinding

A block randomization list with alternating block sizes was generated by the Institute for Medical Biometrics and Informatics (IMBI) using SAS (SAS Version 8.2, SAS Institute Inc., Cary, USA) for each center. The randomization code is stored at the IMBI and is not accessible to any clinical investigator. Until opening the sealed envelopes the allocation sequence is concealed in envelopes. If the indication for a trial randomization is not given after exploration, the sealed envelope will be used for the next patient. Prerequisite for this is an intact seal on the opaque randomization envelope. Envelopes are used because any electronic or telephone systems would have substantially increased the costs of the trial without any funding available to cover these. Furthermore, easily accessible computer systems are not available in all surgical theatres of the participating centers.

Randomization and time measurement is performed by an independent member of the clinical site (study nurse or anesthesiologist) who is not involved in the surgical procedure.

After randomization the assigned group result is concealed. Subsequent investigations are performed by blinded study nurses or blinded investigators in order to assure unbiased assessment of endpoints when possible.

### Statistical analysis

The participating centers collect the data and send the case report forms to the SDGC in Heidelberg. Double data entry is done and queries are generated for statistical monitoring. The analysis will be performed by the IMBI in cooperation with the SDGC using an analysis plan.

A confirmatory analysis of variance (ANOVA) is planned for the primary endpoint based on the intention-to-treat (ITT) principle to test at first the hypothesis H_0_: μ_lig_-μ_clip_≤0 and in case of statistical significance the hypothesis H_0_: μ_lig_-μ_clip_≤15, with the expected operating times μ_lig _and μ_clip_, each on the α-level of 5%. The a priori ordered hypotheses hold the overall α-level of 5%. A randomized patient belongs to the ITT population after the upper vessels have been cut. The fixed factors of this ANOVA will be vessel occlusion technique, center and surgeons experiences. The relation between applied technique and duration of procedure is severely influenced by the surgeon's expertise. Therefore experience is categorized into two groups: surgeons having performed ≥ 50 or <50 thyroid operations. Since there are so far no publications on this topic, the cut-off value of 50 operations was chosen, based on the results of an internal pilot study of the University of Heidelberg on learning curves of surgeons,.

Even if neither superiority nor relevance can be shown statistically, the exploratory interpretation of the narrow two-sided confidence intervals (expected width <10 minutes) can be used to compare the difference in operating time. Some sensitivity analyses are planned, using a linear mixed model as well as using the per-protocol population which consists of all patients of the ITT population without major protocol violations.

Secondary endpoints will be analyzed and characterized using descriptive statistics. An interim analysis is not planned.

### Trial organization, quality control, registration and ethical aspects

The trial was designed at the Study Center of the German Surgical Society (Studienzentrum der Deutschen Gesellschaft für Chirurgie, SDGC) in cooperation with the Institute of Medical Biometrics and Informatics (IMBI) at the University of Heidelberg, Medical School. Trial monitoring is done by an independent monitor of the SDGC. The trial is performed according to the Declaration of Helsinki in its current German version and the Good Clinical Practice (GCP). Before trial start the independent ethic committee gave a positive vote on January 7^th^, 2004. All participants will be informed about the trial and the written informed consent must be signed before randomization. The trial was registered in December 2003 at the International Standard Randomized Controlled Trial Number Registration (ISRCTN96901396).

Two Amendments were accepted before this publication by the ethical committees: in the first amendment two additional secondary objectives (total time of the procedure, weight of specimen) were added. This was done in order to improve evaluation of the primary objective 'resection time' which is calculated by measuring the time between the completion of ligation of the upper pole vessels and the time of removal of the complete specimen. The second amendment was introduced to expand the inclusion criteria in regard to the type of operation. Until then, only hemi-thyroidectomies in combination with subtotal thyroid resection of the other side were included into the trial. Since this operation is not the only standard surgical procedure in goiter surgery in Germany, complete thyroidectomy and near-total thyroidectomy were added. The surgical details of these techniques are not in conflict with the objectives of the study. By this second amendment a shorter recruitment period is expected.

### Current status and duration of the trial

Up to now six centers are initiated and 155 patients, of the 400 needed, were randomized by five centers (status July 5^th^, 2006, details see acknowledgements). The first patient was randomized on March 5^th^, 2004. The expected recruitment period will last until the end of 2008.

## Discussion

The evaluation of optimal surgical therapeutic options and procedures should be performed according to the best current knowledge. Due to the principles of Evidence-based Medicine (EbM), the randomized controlled trial (RCT) is assumed to be the gold standard in evaluating different medical methods and procedures. Even in RCTs, though, the risk of bias should not be underestimated. In surgical trials a double blinded trial design is difficult to establish; therefore, the patient selection, power, randomization, surgeon's skill, data assessment and documentation of outcome measures and statistical analyses are important design issues and susceptible to the different forms of bias. Hence, all these issues are specified in the trial protocol a priori.

Selection bias. To minimize the selection bias all patients scheduled for thyroid surgery are screened consecutively in all participation sites. Moreover, neither patient age nor the size of the goiter is an exclusion criterion. Screening lists and case report files are monitored by independent investigators from the SDGC to ensure correct patient recruitment and meticulous documentation.

Randomization bias. Randomization is done in a block randomization system with alternating block sizes to avoid positive prediction of the last procedures in each block. The calculated randomization sequence is sealed at the IMBI for each participating center. Envelopes are nontransparent and cannot be resealed after opening. After exploration of the thyroid gland and affirmation of the indication an intra-operative randomization is performed. The unclosed randomization sheet is sent via fax to the IMBI.

Surgeon's expertise bias. Another source of bias is the surgeon's expertise. Most often new surgical techniques or methods are introduced by the most experienced surgeons. To minimize this source of bias stratification for surgeon's skill is made, and measured data will be analyzed by analysis of variance in regard to surgeon's skill, center and vessel occlusion technique. Due to this analysis of variance, bias of center effects and surgeon's skill should be reduced. Possible complications such as bleeding, re-operation due to bleeding, recurrent palsy or impaired function of parathyroid glands are assessed by blinded study nurses or investigators. Re-operation, recurrent palsy and impaired function of parathyroid glands are obvious events that can not be diminished by the responsible surgeon.

Prevention of non-conclusive results. Recent reports comparing different methods of hemostasis in thyroidectomies were underpowered or not designed as RCTs. In the calculation of the sample size of 420 patients, a post randomization drop out of 10 patients in each allocation arm and additional 40 patients for each secondary endpoint for 'lost to follow up' are included. Thus, the calculated sample size is large enough to draw definite conclusions. The interpretation of the hypothesis has a high statistical power: a significant result of this relevance test is equivalent to a time difference of at least 15 minutes [19].

Publication bias. One important tool to avoid bias in clinical trials is the international registration and publication of trial protocols. CLIVIT was registered in 2003 before the subsequent requirement of the International Committee of Medical Journal Editors were published [20].

In conclusion, this is the first randomized controlled multicenter relevance trial comparing different vessel occlusion techniques in thyroid surgery with adequate power and sufficient detailed information about the design as well as framework. If significant, the results might be generalized and may change the current surgical practice.

## Competing interests

The author(s) declare that they have no competing interests.
